# Supplementary material for: Cis-perturbation of cancer drivers by the HTLV-1/BLV proviruses is an early determinant of leukemogenesis
Source: Nat Commun. 2017 May 23;8:15264. doi: 10.1038/ncomms15264 (PMC5457497; doi:10.1038/ncomms15264)
Supplement: Supplementary Information — Supplementary Figures, Supplementary Tables and Supplementary References [file ncomms15264-s1.pdf]

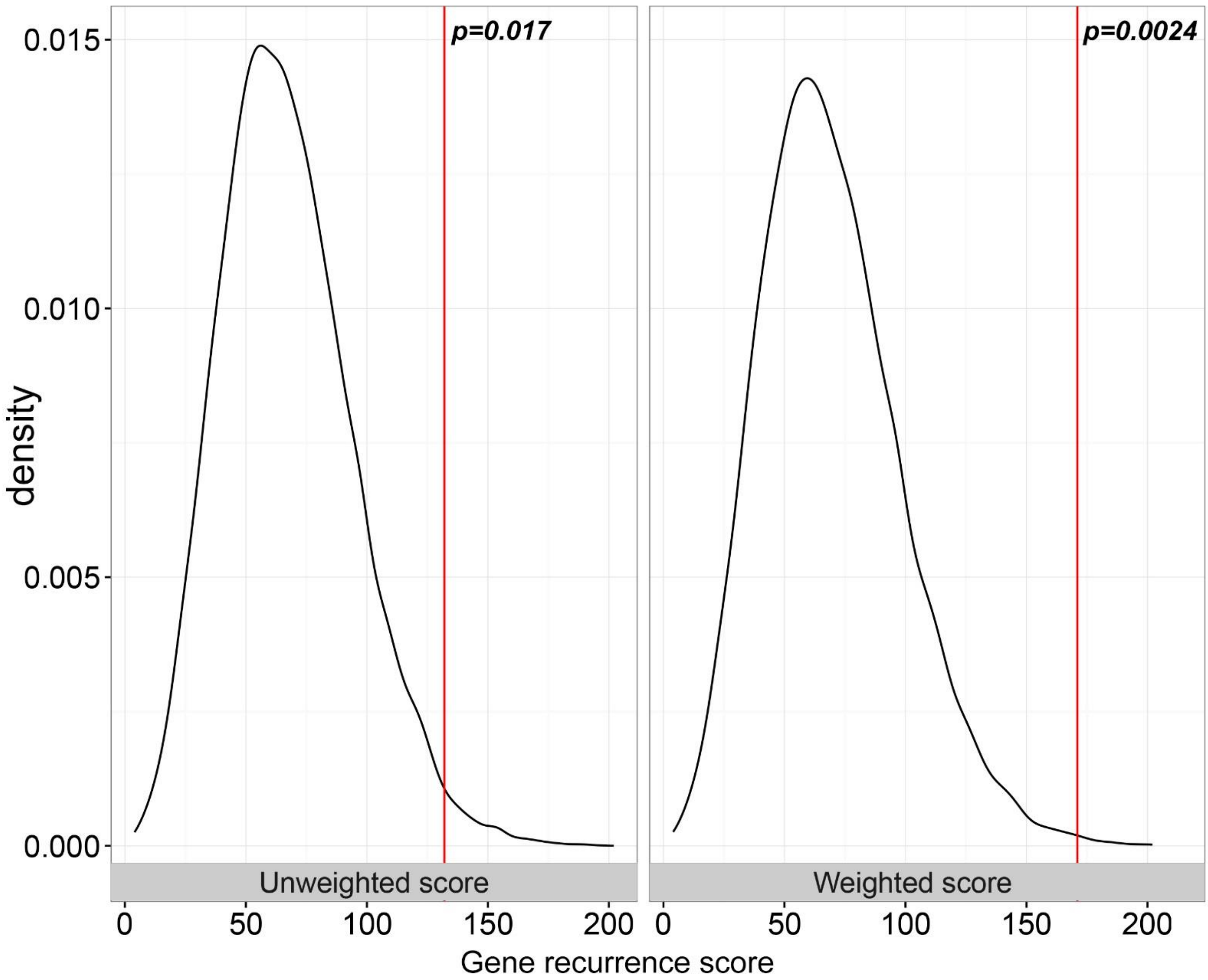

Supplementary Figure 1

Provirus integration sites are biased towards specific regions of the tumor genome: significant gene recurrence in the vicinity of proviral insertions across tumors

Protein-coding genes located within a 1 Mb genomic window upstream and downstream of each distinct tumor proviral integration site (IS, total of 92 sites) were identified and examined for recurrence between tumors. Unweighted and weighted global recurrence scores were obtained by summing individual gene recurrence scores. A gene's unweighted score = 1 regardless of the number of occurrences of that gene across the 92 IS window-based gene lists; a gene's weighted score = the number of occurrences of that particular gene across the 92 IS window-based gene lists. Observed scores were tested against N=100,000 simulated scores obtained from 92 random sets of adjacent genes of same size distribution. Graphs show the distribution of unweighted (left) and weighted (right) scores across the simulated lists. The red line marks the position of the tumor-related real scores. *p* unweighted = 0.017, *p* weighted = 0.0024.

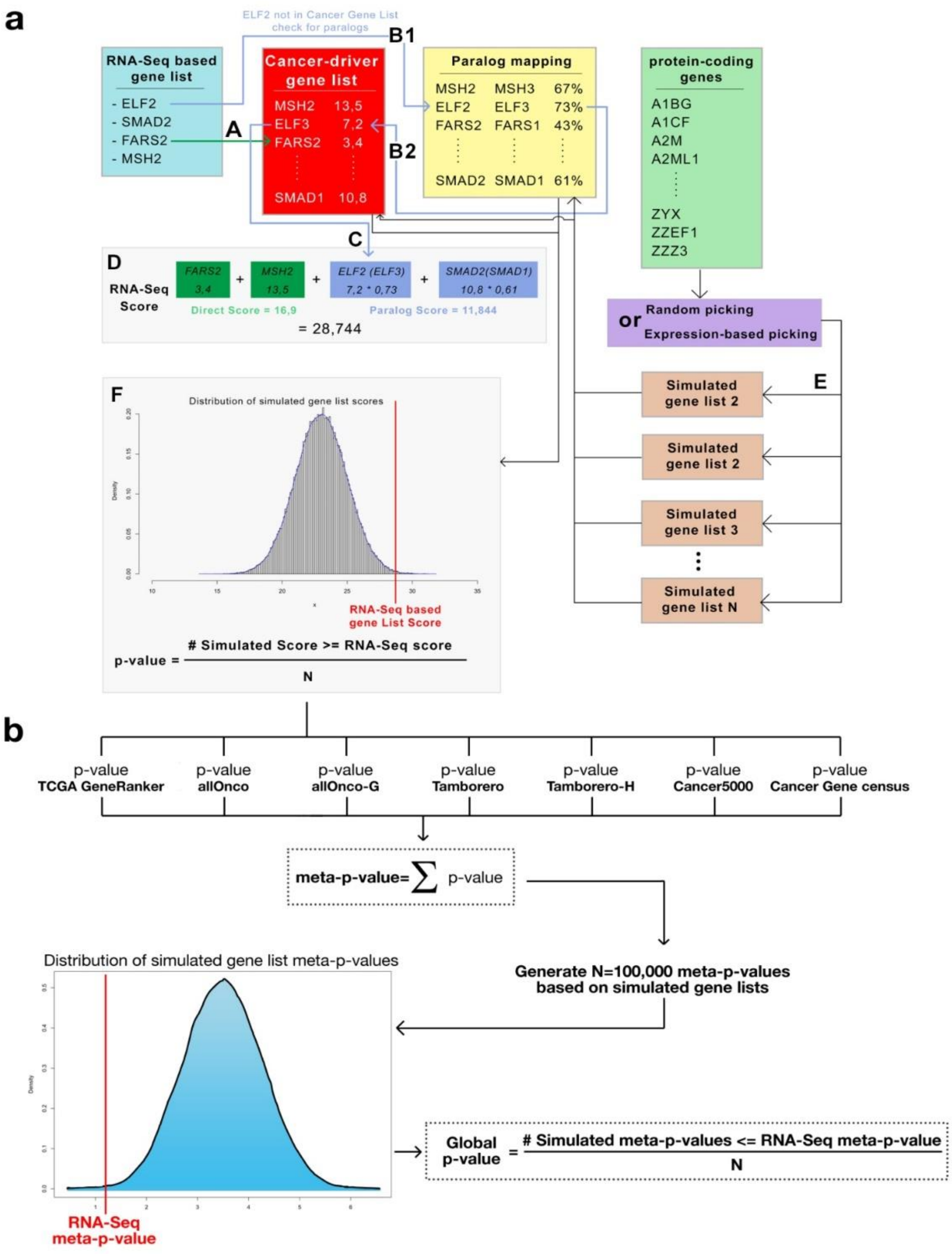

Supplementary Figure 2

Cancer driver enrichment analysis workflow

a. Host gene lists were analysed for enrichment in cancer driver genes as follows: we calculated a total cancer driver enrichment score based on a master set of 7 cancer driver gene lists (CGL) and compared the observed score to simulated scores obtained using a panel of size-matched simulated gene lists. For each gene, we computed a direct score by (A) direct search within the CGLs (i.e. *FARS2* direct score = 3.4, or (B1) paralog search as follows: if a gene is absent from the CGL, a paralog score is computed using the Paralog Mapping Table (ENSEMBL) and (B2) search in the CGL. (C) i.e. *ELF2* paralog score = percentage amino acid identity (*ELF2*-*ELF3*) * Score (*ELF3*). (D) A total score is obtained by computation of the scores of each associated gene. (E) We conducted iterative simulations (N=100,000) of size-matched random or expression-matched gene lists and calculated their associated scores. (F) We compared the observed total score to the N=100,000 simulated scores and computed a *p*-value reflecting the gene set enrichment in cancer-drivers (Methods). **b.** We performed a cancer driver enrichment meta-analysis by computing a meta-p-value (sum of the seven CGL-associated p-values) and comparing this p-value to N=100,000 simulated gene list meta-p-values. A detailed description of the 7 publicly-available cancer driver gene lists and associated score calculation methods are provided in Supplementary Table 2.

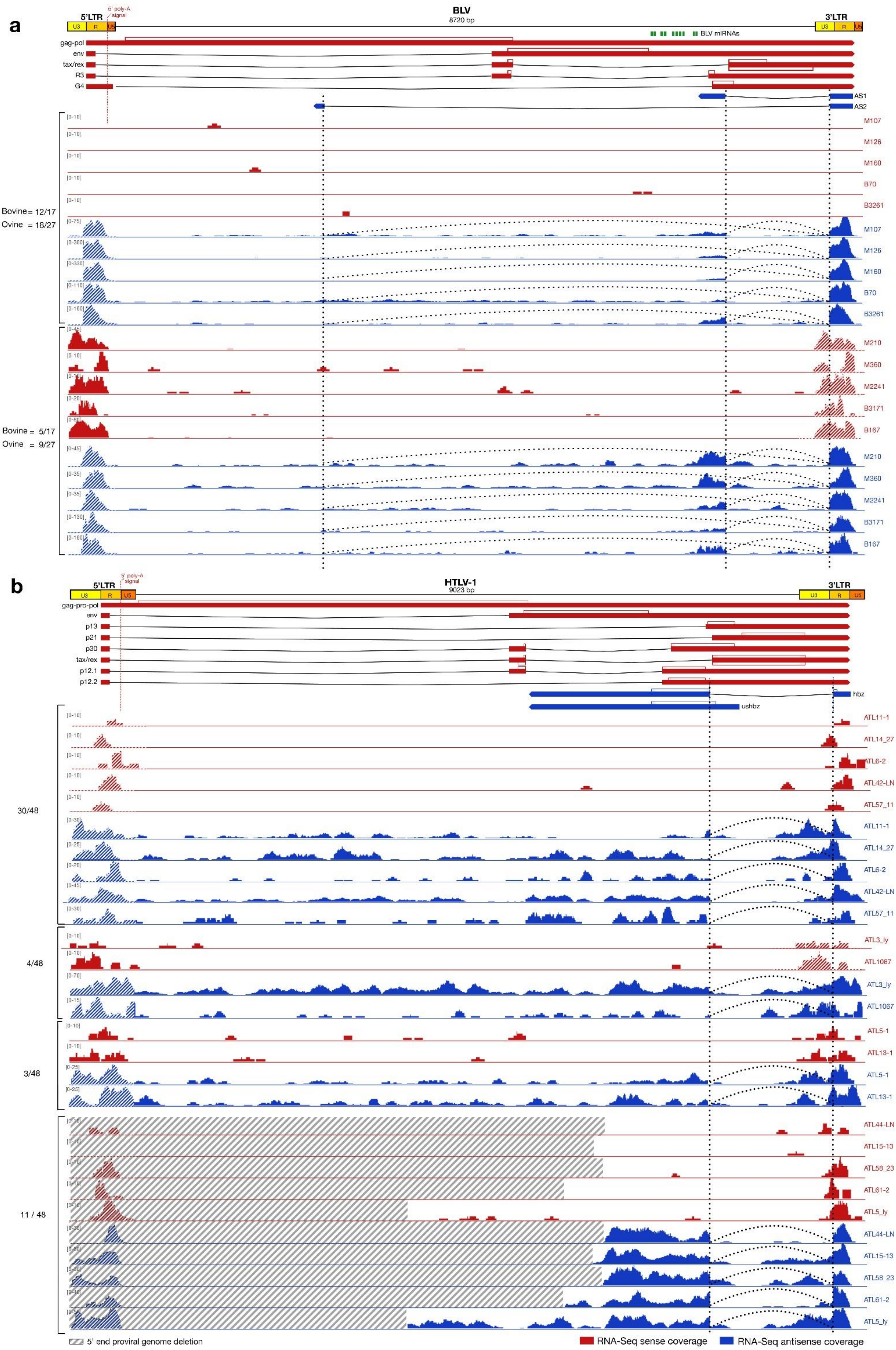
a
BLV
8720 bp
5'LTR
5' poly-A signal
3'LTR
U3
R
U5
BLV miRNAs
gag-pol
env
tax/rex
R3
G4
AS1
AS2
Bovine = 12/17
Ovine = 18/27
Bovine = 5/17
Ovine = 9/27
M107
M126
M160
B70
B3261
M210
M360
M2241
B3171
B167
b
HTLV-1
9023 bp
5'LTR
5' poly-A signal
3'LTR
gag-pro-pol
env
p13
p21
p30
tax/rex
p12.1
p12.2
hbz
ushbz
30/48
4/48
3/48
11 / 48
ATL11-1
ATL14_27
ATL6-2
ATL42-LN
ATL57_11
ATL3_ly
ATL1067
ATL5-1
ATL13-1
ATL44-LN
ATL15-13
ATL58_23
ATL61-2
ATL5_ly
5' end proviral genome deletion
RNA-Seq sense coverage
RNA-Seq antisense coverage

Supplementary Figure 3

Stranded RNA-seq coverage of ATLs and B-cell tumors mapped to the proviral genome

Representative stranded RNA-seq coverages from (a) 10 B-cell tumors, and (b) 14 ATLs mapped to the BLV and HTLV-1 reference genomes respectively and visualized in IGV[1]. Upper panels show the BLV (a) and HTLV-1 (b) proviral genomes and annotations. Viral transcripts encoded from the positive and negative strand coloured red and blue respectively. Open boxes: protein-coding sequences. **a**. RNA-seq coverage of 10 B-cell tumors reveals the complete absence of positive-strand coverage (upper group of 5 tumors representative of 12/17 bovine and 18/27 ovine tumors respectively), or robust positive-strand coverage limited to the 5'LTR U3/R region up to the viral poly (A) signal (bottom group of 5 tumors representative of 5/17 bovine and 9/27 ovine tumors respectively) while negative-strand coverage (blue) is abundant in all examined tumors consistent with the production of 3' LTR-dependent *AS1/AS2* transcripts and additional 3'AS-host hybrid transcripts exposed by coverage extending over the 3' proviral region beyond the annotated *As* 3' end, **b**. RNA-seq coverage of 14 representative ATLs showing the complete absence of positive-strand coverage of protein-coding regions accompanied by low 3'LTR-mapping coverage suggesting a weak 3'LTR sense promoter activity (upper group of 5 ATLs representative of 30/48 proviruses), or robust positive-strand coverage limited to the 5'LTR U3/R region up to the viral poly(A) signal (second group of 2 ATLs representative of 4/48 proviruses). Three proviruses showed evidence of both 3' and 5' LTR positive-strand coverage consistent with both viral poly(A)-dependent polyadenylation of the interrupted host gene and low 3' LTR sense promoter activity (third group of 2 ATLs representative of 3/48 proviruses). In the last group of 5 ATLs (representative of 11/48 proviruses) positive-strand coverage reveals extensive deletions of the HTLV-1 5' region encompassing the 5'LTR. Negative-strand coverage is abundant in all examined ATLs, consistent with the production of 3'LTR-dependent *HBZ* transcripts and additional 3'AS-host hybrid transcripts exposed by antisense coverage extending over the 3' proviral region beyond the annotated *HBZ* 3' end. The 5'LTR-deleted subset of proviruses are characterized by abundant negative-strand coverage of their 3' region that is interrupted beyond the deletion breakpoint. RNA-Seq reads stemming from any of the two LTRs will systematically map to both the 5' and 3' LTR as they are identical in sequence. The LTR from which the coverage specifically derives is indicated by the dark-coloured coverage (red or blue for sense and antisense respectively) while the other LTR is depicted by light-coloured coverage. Reads were assigned to either the 5' or the 3' LTR based on additional virus- and host-specific RNA-seq mapping data (Methods). Note: we failed to detect RNA-seq coverage mapping to coding sequences of full-length tumor proviruses in all but 3 bovine tumors (1351, 3155 et 3261) and 1 ATL (ATL8_9) respectively in which we observed minimal positive coverage that could be assigned to the presence of a minor subpopulation of non-transformed cells.

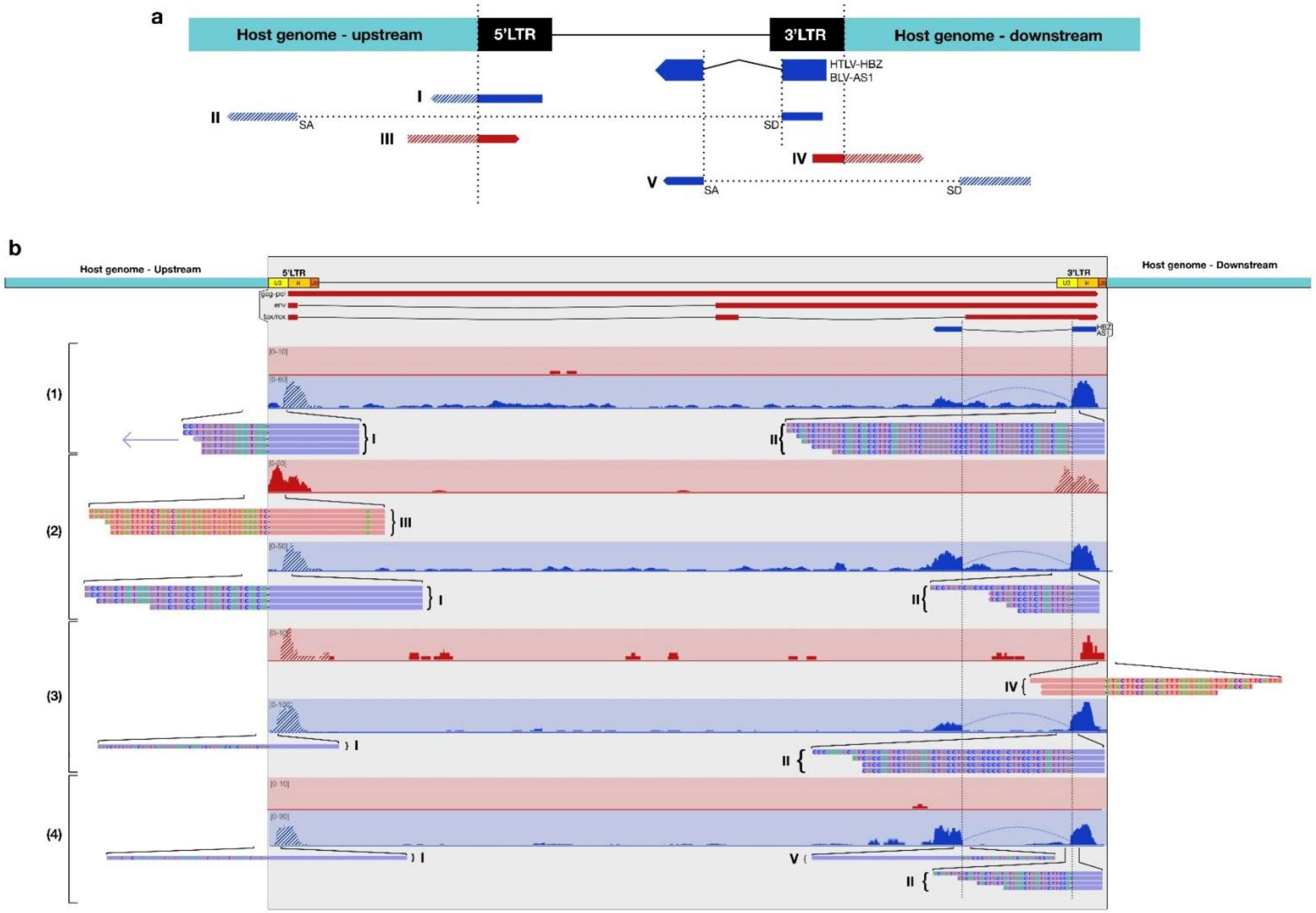

Supplementary Figure 4 Mapping of virus-host hybrid RNA-seq reads in tumors reveals dominant and secondary types of virus-host interactions
a. Virus-host hybrid read classes identified in tumors. Chimeric reads mapping to the provirus positive and negative strand are shown in red and blue respectively. Filled and dashed bars represent provirus and host genome mapping sequences respectively. I: LTR-host and II: *HBZ/AS* exon 1-host hybrid reads reveal unspliced and spliced forms of 3'AS-host chimeric transcripts, III: host-5'LTR hybrid reads reveal host gene transcription (genic-concordant proviruses) through the LTR region and viral poly(A) dependent transcript interruption, IV: 3'LTR-host hybrid reads reveal 3'LTR bi-directional promoter activity resulting in 3'sense-dependent chimeric transcripts (3'S) of low abundance in 14/74 tumors (see also Supplementary Fig. 6), and V: host gene exon (SD) - *HBZ/AS1* exon 2 (SA) hybrid reads reveal the sequestration of *HBZ/AS1* exon 2 by the host gene transcript in genic-discordant proviruses (10/27; Fig. 2b) and potential gene disruption by premature truncation at the *AS1* exon 2 poly(A) site. **b.** RNA-seq coverages 1 – 4 represent possible schemes in tumors. Schemes (2) and (4) were observed in genic-concordant and discordant provirus classes only. Some proviruses combined schemes 2 and 3. Hybrid read classes I and II were systematically observed in all examined tumors. For the 6 integrations in gene deserts, there was no evidence of transcriptional interaction with an annotated gene despite the production of 3'AS dependent hybrid transcript and upstream genomic RNA-Seq coverage.

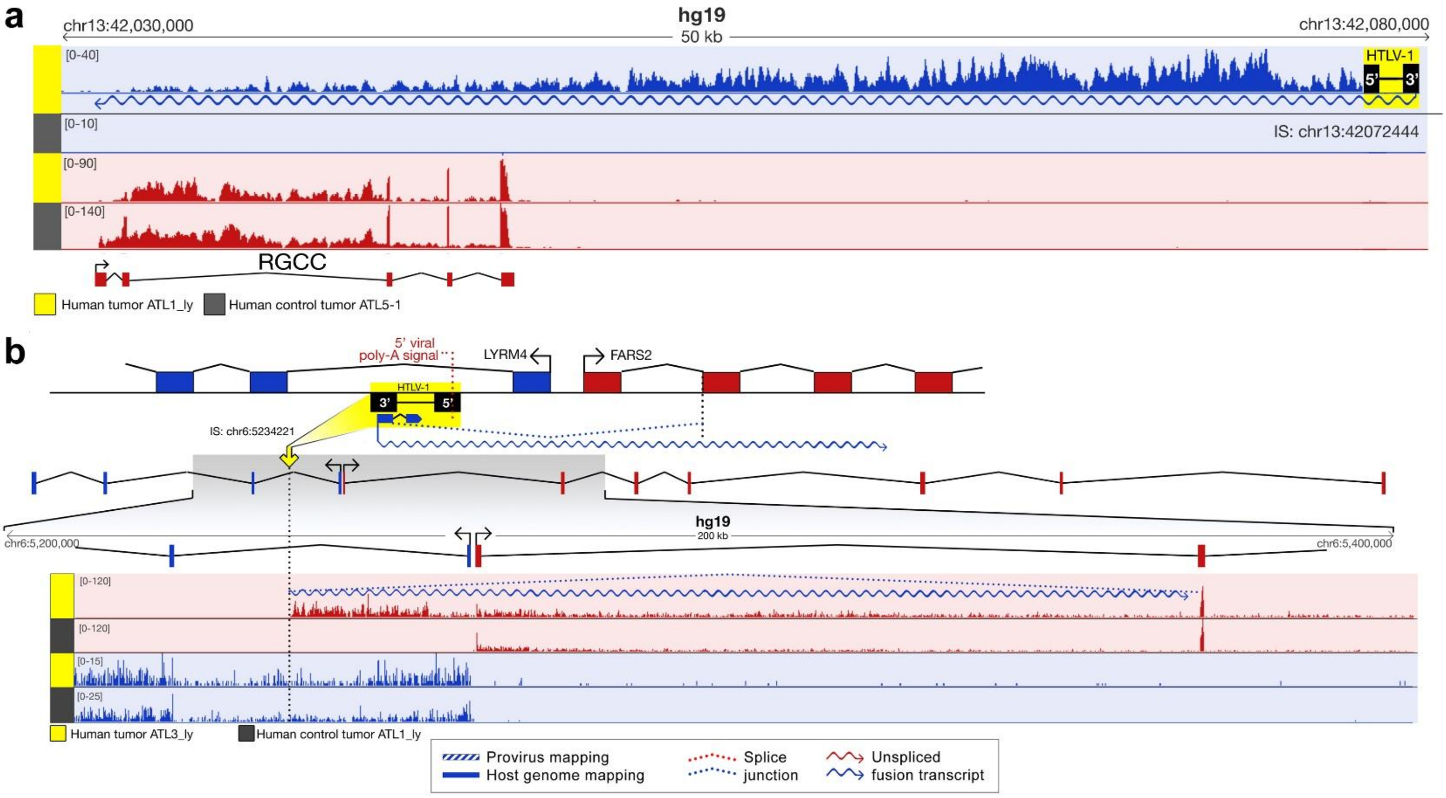

Supplementary Figure 5

Viral antisense RNA-dependent cis-perturbation of host genes in representative tumors (see also Fig. 3).

a. Intergenic concordant provirus: Intergenic-concordant provirus and 3’AS-dependent antisense gene overlap in human leukemia ATL1-Ly. HTLV-1 integration downstream of *RGCC* (Regulator of cell cycle) and expression of a 3’AS-dependent hybrid transcript in antisense overlap with *RGCC*.

b. Genic concordant provirus – interaction with multiple genes: Combination of viral antisense- and poly-(A)-dependent perturbations of adjacent genes transcribed from opposite DNA strands. Human leukemia ATL3_Ly: genic-concordant HTLV-1 provirus integrated within *LYRM4* (yellow arrow) causes a second interaction with the adjacent gene (*FARS2*) by sense overlap of a 3’AS-dependent hybrid transcript and additional capture of *FARS2* exon 2.

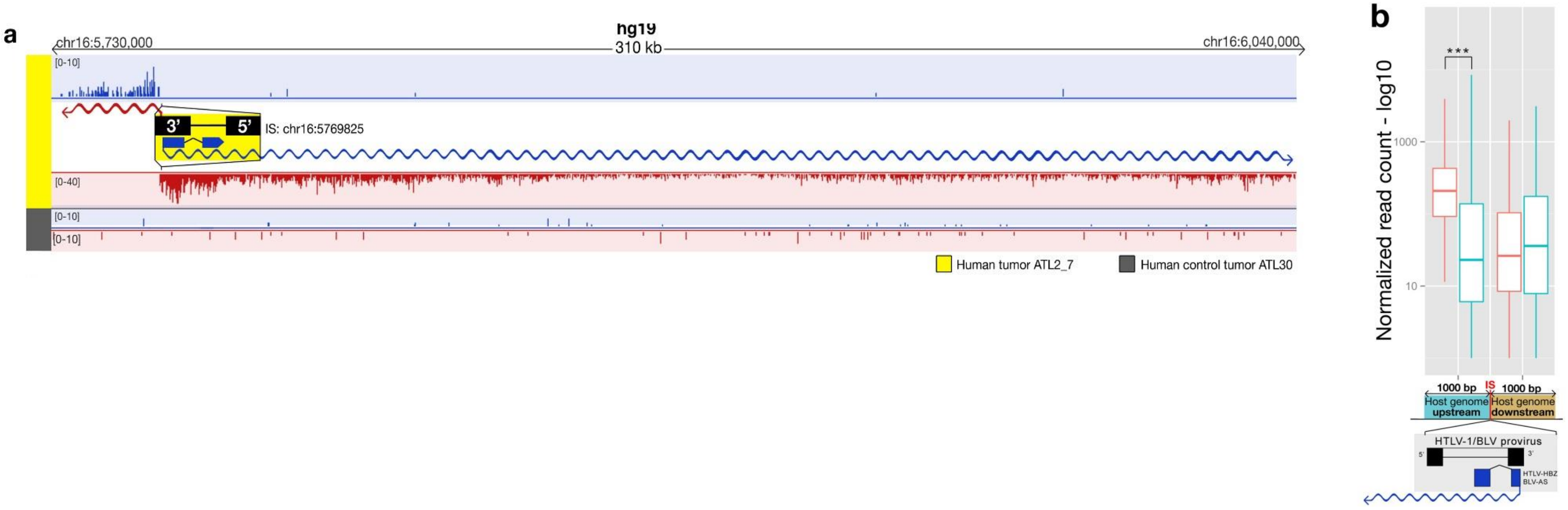

Supplementary Figure 6

Antisense-predominant transcriptional activity of the viral 3'LTR and upstream host genomic regions is accompanied by a significantly weaker sense activity in 14 tumors

a. Representative example of a tumor provirus showing bi-directional 3'LTR promoter activity. In ATL2_7 intergenic-discordant HTLV-1 (chr16:5769825) exhibits both 3'AS-dependent (curved blue line, host genome plus-strand) and 3'S-dependent (curved red line, host genome minus-strand) hybrid transcripts. Control ATL30 (black bars). **b**. Transcriptional activity of upstream and downstream flanking host genomic regions relative to tumor-associated proviral integrations. Normalized RNA-Seq coverage of a 1000 bp genomic window upstream (left panel) or downstream (right panel) of the tumor-associated viral integration sites in tumors with integrations of interest (red dot plots), or control tumors without provirus integration at these genomic locations (blue dot plots) *** p-value ≤ 1e-35, Mann Whitney U test.

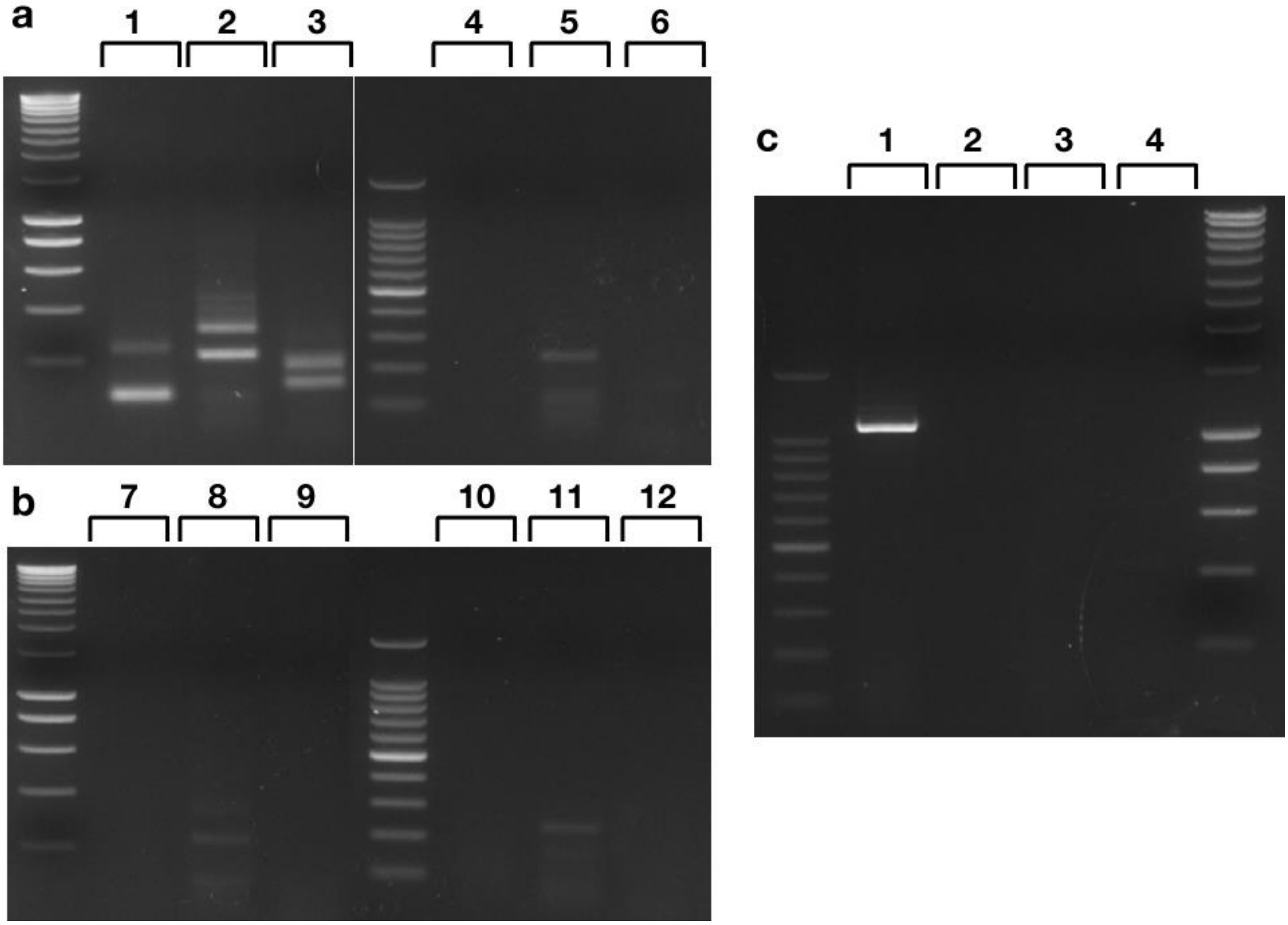

Supplementary Figure 7

Validation of chimeric transcripts by RT-PCR

RT-PCR confirmation of spliced virus-host chimeric transcripts revealed by RNA-seq-based identification of split reads and splice junctions. Primers sequences in Supplementary Data 5. **a.** and **b.** RT-PCR products exposing BLV-host chimeric transcripts that result from the sequestration of BLV *As* exon 1 splice donor (discordant proviruses) by host gene exon splice acceptors in *ELF2* exon 3 (YR2 and M395 tumor, lane 1, 116 bp), *PRPSAP2* exons 1 and 2 (M28, lane 2, 228 and 326 bp) and *SEPT11* exon 2 and a novel exon upstream of *SEPT11* exon 1 (LB120, lane 3, 147 and 208 bp). Lanes 4-6: corresponding RT minus products. Lanes 7 – 12: products obtained using RNA from control tumor B70 with primers and conditions as described in 1-6. 100 bp ladder (Promega) **c.** RT-PCR product revealing BLV-host chimeric transcript that result from the sequestration of BLV *AS* exon 1 splice donor (concordant provirus) by a cryptic host genome splice acceptor site in *MYCBP2* (tumor M2531, lane 1, RT +, 1079 bp; lane 2, RT-). Lanes 3-4: products obtained using RNA from control tumor B70 with primers and conditions as described in 1-2. Ladder: smart ladder 200 bp-10 kb (Eurogentec).

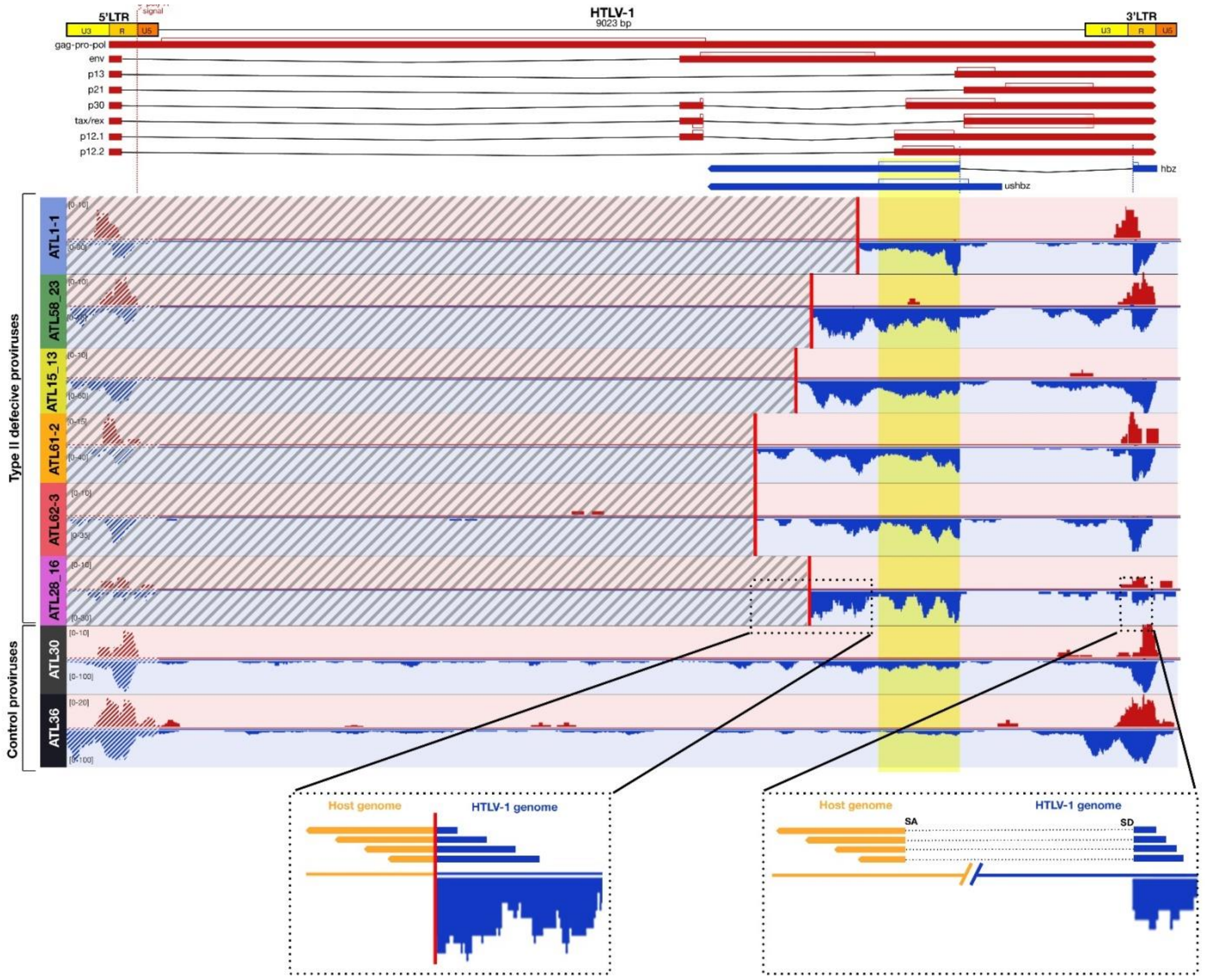

Supplementary Figure 8

5'LTR-deleted defective HTLV-1 proviruses identified in ATLs produce 3'AS-dependent chimeric antisense transcripts that involve upstream host genes

HTLV-1 RNA-seq coverage of 6 ATLs characterized by a single predominant 5'LTR-deleted provirus (11/38 ATLs, clonal abundance 96.75 – 99.9 %, Supplementary Data 2) reveals non-canonical virus-host boundaries and absence of both sense (red) and antisense (blue) coverage upstream of this non-canonical virus-host breakpoint. Deletions range from 5618 to 6440 kb (dashed region) and result in irreversibly impaired mRNA production from the positive strand. Lower panel (boxes): identification of hybrid reads that expose the virus-host breakpoint (left box) and *HBZ* exon 1-dependent splicing to host genomic sequences (right box), revealing the production of chimeric transcripts that affect upstream host genes. In ATL28-16, the defective HTLV-1 provirus produces a 3'AS-dependent transcript that captures exon 3 of *SPSB1*, a negative regulator of the TGF-ß pathway the interruption of which was found associated with tumor progression and metastasis[2]. The *HBZ* protein-coding sequence (yellow region) is retained in all ATLs that carry defective HTLV-1. Upper panel: HTLV-1 genome and annotation (positive strand-encoded viral mRNAs colored in red, spliced/unspliced *Hbz* transcripts colored in blue). ATLs that carry a full-length HTLV-1 provirus exhibit antisense RNA-seq coverage over the total length of the proviral genome (ATL30 and ATL36, black bars).

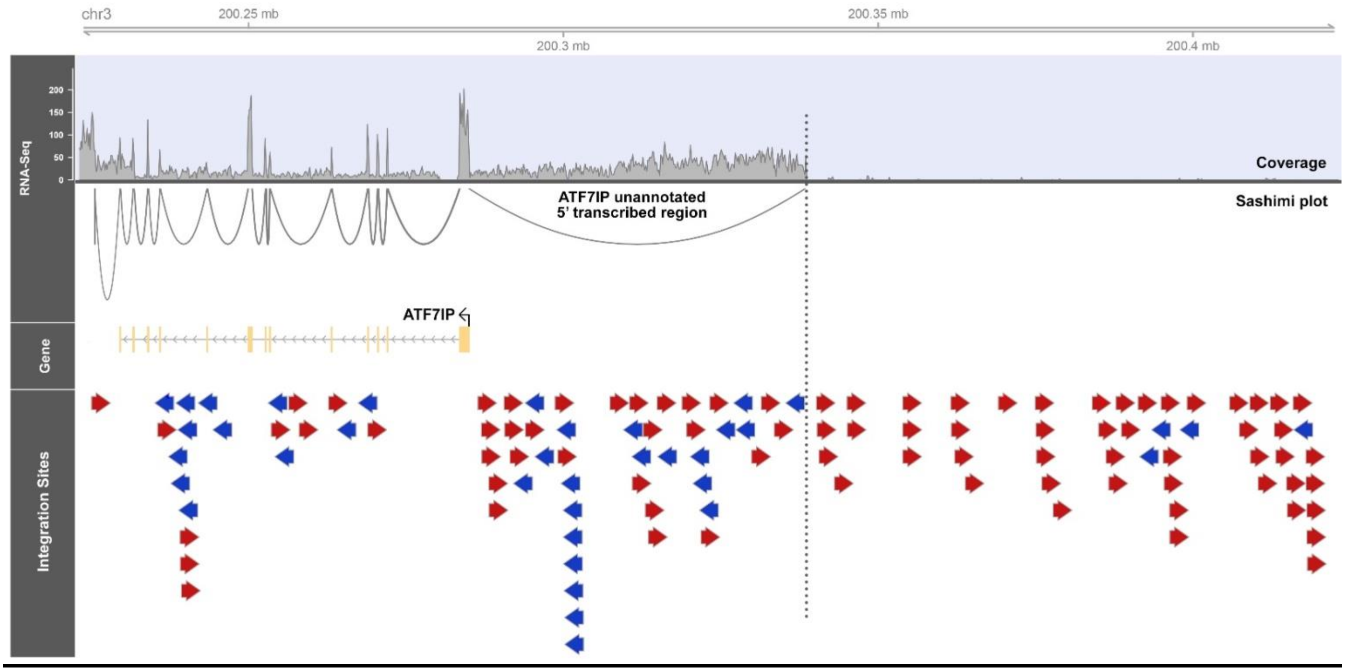

Supplementary Figure 9

Mixed integration hotspot that combines genic and intergenic integration sites.

Proviral integration sites (arrows indicate provirus position and orientation relative to the reference genome considering 5’ – 3’) in the vicinity of *ATF7IP*[3] and corresponding RNA-seq coverage shown in IGV. 120 integration sites (orientation ratio same/opposite in favour of positive orientation: 0.7) were found distributed between the annotated genic region, the 5’ transcribed yet unannotated genic region (ENSEMBL OAR3.1 v84, and the intergenic non-transcribed region upstream of *ATF7IP*.

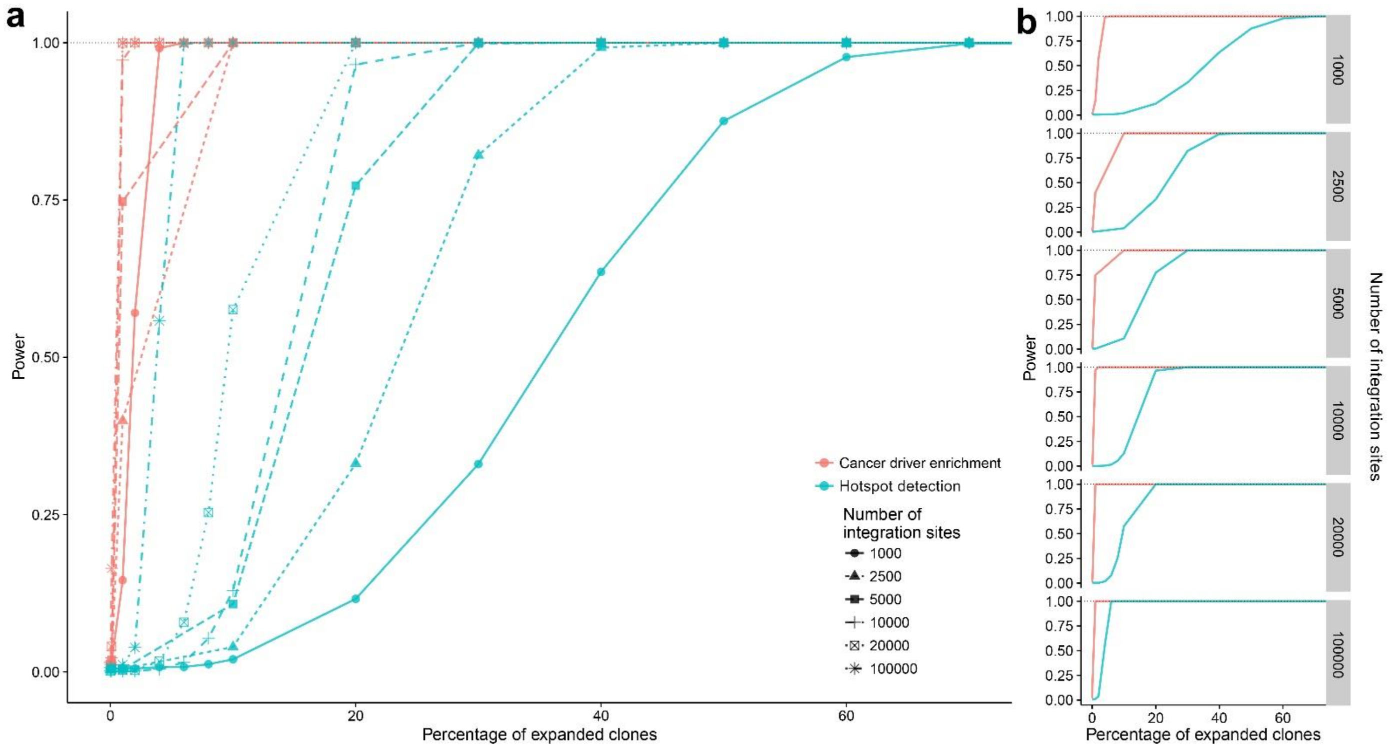

Supplementary Figure 10

Statistical power of cancer driver enrichment analysis is superior to that of hotspot identification

To test the power of the cancer driver enrichment and integration hotspot detection statistical tests, we performed simulations assuming that a fraction w of x proviral integration sites are sampled from clones that are undergoing expansion due to perturbation of one of y cancer drivers (out of a total of 20,000 genes), of which a fraction z is reported in cancer driver lists. The remaining fraction (1-w) of proviral integration sites are assumed to be sampled from infected but non-expanded leucocyte clones. w was varied from 0.0001 to 1, x from 1,000 to 100,000, y from 500 to 3,000, and z from 0.25 to 1 (see *Methods*). The statistical test for enrichment in cancer drivers (red curves) was considerably more powerful than that for the detection of integration hotspots (blue), for all parameter combinations, shown here for y = 1,000, z = 1, and varying numbers of IS (1,000 to 100,000) and percentages of expanded clones (0.01 to 100 %) respectively. **a.** Statistical power for all IS numbers ranging from 1,000 to 100,000. **b.** Statistical power for each IS number considered individually

Supplementary Table 1. Characterization of proviral integration sites in human ATLs (HTLV-1) and ovine/bovine B-cell tumors (BLV)

**Proviral integration site genomic environment**										
	**Genic**				**Intergenic**				**Tot**	**%**
	**Ovine**	**Bovine**	**Human**	**Tot**	**Ovine**	**Bovine**	**Human**	**Tot**		
**Concordant gene-provirus orientation**	11	5	11	**27**	0	2	6	**8**	**35**	**38.04**
**Discordant gene-provirus orientation**	10	5	12	**27**	6	3	15	**24**	**51**	**55.43**
**Gene desert**	n/a	n/a	n/a	**n/a**	0	2	4	**6**	**6**	**6.52**
**Total**	**21**	**10**	**23**	**54**	**6**	**7**	**22**	**38**	**92**	
**%**				**58.7**				**41.3**		

**Proviral integrations (number/tumor)**							
	**1 IS**	**2 IS**	**3 IS**	**4 IS**	**7 IS**	**Tot**	**IS number**
**Ovine**	**25**	**1**	**0**	**0**	**0**	**26**	**27**
**Bovine**	**12**	**1**	**1**	**0**	**0**	**14**	**17**
**Human**	**28**	**3**	**1**	**1**	**1**	**34**	**48**
**Total**	**65**	**5**	**2**	**1**	**1**	**74**	**92**

Genomic environment of tumor proviral integration sites (IS) as determined by RNA-Seq chimeric read detection combined with HTS integration mapping of proviral integration sites. 100 integration sites were identified across all tumor samples, defining a list of 92 distinct integration sites (identical sites identified in different samples from the same individual - lymphoma, leukemia, treatment - were merged). Genic/intergenic: provirus position relative to host gene. Concordant/discordant: provirus transcriptional orientation relative to host gene (assuming predominant 5'LTR to 3'LTR transcription). Reference genomes hg19 (human), UMD3.1 (bovine) and OAR3.1 (ovine). Gene annotations according to ENSEMBL v84. Gene desert: untranscribed host genomic region according to ENSEMBL and RefSeq annotations. n/a: not applicable.

Supplementary Table 2. Publicly-available cancer driver catalogues used for enrichment simulations, associated gene numbers and gene scores

Cancer-driver gene list (CGL)	Gene numbers	Gene scores	Reference
Cancer5000	260	Defined by CGL authors	[4]
Tamborero / Tamborero-H	435/291	1 / 2	[5]
Cancer Gene census	522	1	[6]
AllOnco / AllOnco-G	2129	1 / 1-8	[7]
TCGA GeneRanker	7658	Defined by CGL authors	[8]

CGL: Cancer5000 and Tamborero: high confidence cancer driver candidates established from studies of somatic mutations in thousands of tumors using exome and whole-genome sequencing and supported by robust statistical analysis. Tamborero-H: variant sub-list restricted to the 291 highest confidence drivers from the total set of 435 candidate drivers. “Cancer Gene census”: compilation of genes from the literature that were found mutated and causally implicated in cancer. AllOnco: list of 2129 candidate genes generated from eight published cancer driver lists. AllOnco-G: variant list in which the number of occurrences of a particular gene across the eight cancer driver lists was recorded. TCGA GeneRanker catalogue was established from 40 cancer drivers list (https://tcga-data.nci.nih.gov/tcga).

Gene scores used for enrichment simulations were either (i) well established statistically sound scores devised by the authors of these lists (Cancer5000, TCGA gene ranker), or (ii) if scores were not available (AllOnco, Tamborero, CancerCensus), a score of 1 was reported if the gene was present in the list, or (iii) when combining lists (AllOnco-G, 8 lists), the score reflects in how many of these lists the gene is reported, and (iv) in the “Tamborero-H” list, a score of 2 was reported (the gene is a high confidence driver) while a score of 1 was assigned to a “regular” cancer driver (“Tamborero”).

Supplementary Table 3. Summary of HTLV-1/BLV sample RNA-Seq data and virus-host hybrid read detection.

	Tumor samples				Infected – asymptomatic		Asymptomatic – AS-enriched
	**Ovine**	**Bovine**	**Human**	**Total**	**HTLV-1 AC**	**BLV – ovine**	**BLV – ovine**
Sample number	32	15	44	91	4	10	10
Samples with detected fusion	30	14	36	81	1	10	10
Read counts mean (min-max)	97,964,472 (28,128,996-302,000,000)	110,667,144 (51,027,568-324,000,000)	108,800,346 (29,019,686-242,000,000)	102,750,708 (28,128,996-324,000,000)	126,305,526 (76,608,556-153,708,216)	119,608,290 (6,378,406-302,977,252)	1,334,939 (950,640 – 1,666,612)
% Uniquely mapped reads mean (min-max)	73.59 (58.34-84.29)	83.35 (59.34-92.56)	87.23 (80.05-94.63)	80.19 (58.34-94.63)	88.19 (85.54-91.1)	72.04 (56.04-82.73)	79.18 (54.35-87.01)
Viral antisense TPM mean (min-max)	1.21 (0.06-5.83)	1.195 (0.17-2.76)	1.53 (0.04-14.8)		0.025 (0-0.1)	0.33 (0-0.93)	NA
Chimeric reads AS/HBZ-defined mean (min-max)	24.08 (0-188)	8.84 (0-34)	6.64 (0-74)	13.30 (0-188)	0.5 (0-2)	3.63 (0-10)	48,977 (6,644-114,510)
Chimeric reads LTR-defined mean (min-max)	5.68 (0-56)	12.42 (0-68)	4.58 (0-30)	6.54 (0-68)	0 (0-0)	3.45 (0-8)	1,936.9 (290-4,438)

RNA-seq raw reads were aligned using STAR[9] and host reference genomes hg19 (human), OAR3.1 (ovine), UMD3.1 (bovine), and proviral genome references HTLV-1 ATK-1 (GenBank : J02029) and BLV YR2 (GenBank : KT122858) respectively. TPM (Transcripts Per Million): relative measure of viral antisense transcript abundance (BLV *AS1*, HTLV-1 *HBZ*) computed using RSEM[10]. Chimeric reads were identified using a custom detection pipeline. Tumor samples in which we failed to detect hybrid reads either were sequenced at insufficient depth or harboured low proviral loads (i.e. samples from treated ATL patients that responded to therapy, PBMC samples from lymphoma-affected ATL patients, detailed description of all samples available in Supplementary Data 2). Sequenced samples included both biological and technical duplicates (38 samples: 11, 17 and 10 from ovine, human and bovine origin respectively). Sequencing of libraries prepared following BLV 3'AS-enrichment of RNA from asymptomatic sheep resulted in an increased number of hybrid reads per sample and per chimeric transcript. Individual interacting host genes (hybrid transcripts) were supported by a mean of 279.2 (range of 1-51,470) and 23.7 (range of 1-761) hybrid reads from 3'AS-host and LTR-host fusions respectively.

Supplementary Table 4. HTLV-1/BLV transcript-interacting host gene types identified in tumors.

	PC	PC + lncRNA	lncRNA	PC + NA	Gene desert	Total	%
**Ovine**	25	0	0	2	0	27	29.34
**Bovine**	12	0	0	3	2	17	18.47
**Human**	24	9	11	0	4	48	52.17
**Total**	61	9	11	5	6	92	
**%**	66.30	9.78	11.95	5.32	6.52		

Host gene types interacting with HTLV-1/BLV-dependent virus-host hybrid transcripts for each of the 92 distinct proviral integrations identified in the tumor RNA-Seq datasets as identified by a custom-made RNA-seq based hybrid transcript detection pipeline and additional manual curation of the data using IGV visualization. Valid host genes met the following criteria: (i) the gene was uncovered by the fusion detection pipeline (hybrid reads did involve this gene or upstream/downstream genomic region), (ii) manual examination in IGV and RNA-seq coverage confirmed chimeric transcript production and position, (iii) the host gene transcription pattern was found affected either quantitatively or qualitatively (alternative splicing, exon skipping, creation of novel exons), or the hybrid transcript was found in overlap with an expressed host gene in either orientation, or the hybrid transcript was produced in convergent overlap with a host gene that is not expressed in control samples. (iv) Were added to this list: target host genes that were not revealed by the pipeline yet found interacting upon manual examination in IGV (i.e. multiple adjacent genes affected by the same virus-dependent transcript), and host genes positioned downstream of fusion transcript at the boundary of its RNA-Seq coverage (5 genes). Interacting host genes consisted of protein-coding genes (PC), noncoding RNAs and pseudogenes (lncRNA), and unannotated or unknown host transcripts (NA). A fraction of proviruses (6.52 %) appeared positioned in unannotated regions or gene deserts (GD) and did not directly target known genomic features. Details of host gene interactions for each individual tumor sample are described in Supplementary Data 2.

Supplementary Table 5.

Recurrent provirus-affected host genes between asymptomatic non-malignant samples (sheep, DNA-seq) and tumor samples (3 species)

**BLV asymptomatic sheep – Genic hotspots of proviral integration (674 genes)**<br>Overlap with genes identified by RNA-seq analysis of tumor samples (HTLV-1/BLV, 74 genes) $p = 0.00073$	
**Gene (9)**	**Species**
KLHL14	Ovine
KPNA3	Ovine
LHPP	Ovine
MYCBP2	Ovine
OSBPL8	Ovine
SCAF8	Ovine
STK17A	Human
TCF4	Bovine
TLE4	Human

Supplementary Table 6.

Recurrent provirus-affected host genes between asymptomatic non-malignant samples (sheep, capture RNA-seq) and tumor samples (3 species)

**BLV asymptomatic sheep – Genes revealed by AS-capture RNA-seq (723)**<br>Overlap with genes identified by RNA-seq analysis of tumor samples (HTLV-1/BLV, 74 genes) *p* = 0.00085	
**Gene (9)**	**Species**
DDX10	Bovine
ELF2	Ovine/Human
ICA1	Ovine
KSR1	Ovine
N4BP2	Ovine
NF1	Ovine
SNIP1	Ovine
TCF4	Bovine
TLE4	Human

Supplementary Table 7

Characterization of minor integration sites identified by high-throughput DNA-seq mapping of HTLV-1 insertion sites in 228 cases of ATL

ATL samples	31 (this study)	197[11]
Integration sites (IS)	4,628	11,279
Genic integrations (genes with ≥ 1 HTLV-1 IS)	7,155 (4,500)	
Cancer driver enrichment (genic integrations)	p < 1e-05	
Gene recurrence with genic hotspots identified in asymptomatic sheep (674)	293 genes, p = 2.889411e-13	
Gene recurrence with interacting genes revealed by	41 genes, p = 6.688275e-05	

4,628 HTLV-1 integration sites (IS) were identified by DNA-seq of 31 ATL cases (this study). 11,279 HTLV-1 IS previously identified in 197 cases of ATL by Cook *et al.*[11] were retrieved from the NCI Retrovirus Integration Database (RID), a public database for retroviral insertion sites[12]. Hotspot analysis was performed as described for asymptomatic BLV infected sheep (Methods). Cancer driver gene enrichment of genes showing ≥ 1 IS was performed by simulation (4500 genes, ENSEMBL v84) with N=100,000 iterations. Recurrence between the 4500 HTLV-1 target genes and (i) the genes corresponding to the genic hotspots identified by HTS based IS mapping in BLV infected asymptomatic sheep (674 genes) or (ii) the cis-perturbed genes identified by RNA-seq in malignant clones of the three species (74 genes, Supplementary Table Data 4) was analysed using a one-tailed Fisher's exact test.

The abundance of the corresponding HTLV-1 infected clones was not reported in the public RID.

Supplementary Table 8.

Recurrent genes (41) between the HTLV-1 target genes revealed in 31 ATLs from this study and 197 ATLs from the study by Cook *et al*.[11] and the genes identified in tumor clones (RNA-seq, 74 genes, ATLs and B cell tumors, three species).

**Genes**	**Species**
ARHGEF4	OAR
CA10	HSA
CGGBP1	OAR
COL22A1	OAR
DDX10	BTA
DNMT3A	HSA
DSG2	HSA
ELF2	HSA/OAR
FARS2	HSA
HECTD1	HSA
HUWE1	BTA
ICA1	OAR
KLHL14	OAR
KPNA3	OAR
KSR1	OAR
LCORL	HSA
MAST4	HSA
MCC	HSA
MSH2	BTA
MYCBP2	OAR
NAPG	BTA
NF1	OAR
NUMB	HSA
OSBP	HSA
RASA3	OAR
RBFOX1	HSA
RRAGB	OAR
SCAF8	OAR
SEPT11	BTA
SNUPN	HSA
SPOCK1	BTA
SPSB1	HSA
STK31	HSA
TCF4	BTA
TLE4	HSA
TLR9	HSA
TMEM67	HSA/BTA
TNKS	BTA
UBASH3B	OAR
VPS39	HSA
WDR82	OAR
Overlap: 41 / 74 genes	

Supplementary Table 9.

Integration sites (IS) that match abundant clones in asymptomatic sheep show a higher degree of integration in hotspots and cancer driver enrichment

**Hotspot detection**						
**Threshold read number**	**AB+/HS+**	**AB+/HS-**	**AB-/HS+**	**AB-/HS-**	**Odds Ratio**	***p*-value**
2	14362	19583	12901	19711	1.1205	2.87E-13
5	3294	4013	23969	35281	1.2082	2.26E-14
10	772	724	26491	38570	1.5524	3.00E-17
50	21	11	27242	39283	2.7528	0.004211
100	8	3	27255	39291	3.8442	0.034041

**Cancer driver enrichment**				
	**AB+**		**AB-**	
**Threshold read number**	**Genes**	***p*-value**	**Genes**	***p*-value***
2	4357	<1e-05	4326	<1e-05
5	1345	<1e-05	5630	0.00115
10	424	<1e-05	5736	0.0009
50	17	0.1104	5759	0.2069
100	6	0.1097	5760	0.33165

The abundance of BLV infected clones corresponding to the 66,557 distinct IS identified in asymptomatic sheep was defined by the number of sequencing reads supporting each IS (Methods). Clones were assigned to two classes of abundance (abundant: Ab+ and non-abundant: Ab-) according to increasing thresholds of sequencing read numbers (from 2 to 100 reads per IS, six thresholds each corresponding to a row in the table). For hotspot detection, the genomic location (relative to the 722 hotspots defined by simulation; within a HS: HS+, not in HS: HS-) of abundant and non-abundant clones respectively was defined for each threshold. A one-tailed Fisher's exact test was performed to assess the statistical enrichment of abundant clones in hotspots of proviral integration. Cancer driver enrichment of AB+ and AB- genic IS respectively was performed by simulation using size-matched IS subsets obtained by subsampling of each AB- IS group based on the size of the corresponding AB+ IS group. * median *p*-value obtained by subsampling N=100

References to Supplementary Figures and Tables

1. Thorvaldsdottir, H. *et al.* Integrative Genomics Viewer (IGV): high-performance genomics data visualization and exploration. *Briefings in Bioinformatics* **14,** 178–192 (2013).

2. Liu, S., Nheu, T., Luwor, R., Nicholson, S. E. & Zhu, H.-J. SPSB1, a Novel Negative Regulator of the Transforming Growth Factor-β Signaling Pathway Targeting the Type II Receptor. *J. Biol. Chem.* **290,** 17894–17908 (2015).

3. Waterfield, M. *et al.* The transcriptional regulator Aire coopts the repressive ATF7ip-MBD1 complex for the induction of immunotolerance. *Nat. Immunol.* **15,** 258–265 (2014).

4. Lawrence, M. S. *et al.* Discovery and saturation analysis of cancer genes across 21 tumour types. *Nature* **505,** 495–501 (2014).

5. Tamborero, D. *et al.* Comprehensive identification of mutational cancer driver genes across 12 tumor types. *Scientific reports* **3,** 2650 (2013).

6. Futreal, P. A. *et al.* A census of human cancer genes. *Nature reviews. Cancer* **4,** 177–183 (2004).

7. Sadelain, M., Papapetrou, E. P. & Bushman, F. D. Safe harbours for the integration of new DNA in the human genome. *Nature Reviews Cancer* **12,** 51–58 (2011).

8. Cerami, E. Gene Ranker: TCGA GBM 6000. (2009). at <http://cbio.mskcc.org/tcga-generanker/index.jsp>

9. Dobin, A. *et al.* STAR: ultrafast universal RNA-seq aligner. *Bioinformatics* **29,** 15–21 (2013).

10. Li, B. & Dewey, C. N. RSEM: accurate transcript quantification from RNA-Seq data with or without a reference genome. *BMC bioinformatics* **12,** 323 (2011).

11. Cook, L. B. *et al.* The role of HTLV-1 clonality, proviral structure, and genomic integration site in adult T-cell leukemia/lymphoma. *Blood* **123,** 3925–3931 (2014).

12. Shao, W. *et al.* Retrovirus Integration Database (RID): a public database for retroviral insertion sites into host genomes. *Retrovirology* **13,** 47 (2016).
